# The “handedness” of language: Directional symmetry breaking of sign usage in words

**DOI:** 10.1371/journal.pone.0190735

**Published:** 2018-01-17

**Authors:** Md. Izhar Ashraf, Sitabhra Sinha

**Affiliations:** 1 The Institute of Mathematical Sciences, Chennai, Tamil Nadu, India; 2 B. S. Abdur Rahman University, Chennai, Tamil Nadu, India; 3 National Institute of Advanced Studies, Bengaluru, Karnataka, India; University of Maryland, UNITED STATES

## Abstract

Language, which allows complex ideas to be communicated through symbolic sequences, is a characteristic feature of our species and manifested in a multitude of forms. Using large written corpora for many different languages and scripts, we show that the occurrence probability distributions of signs at the left and right ends of words have a distinct heterogeneous nature. Characterizing this asymmetry using quantitative inequality measures, viz. information entropy and the Gini index, we show that the beginning of a word is less restrictive in sign usage than the end. This property is not simply attributable to the use of common affixes as it is seen even when only word roots are considered. We use the existence of this asymmetry to infer the direction of writing in undeciphered inscriptions that agrees with the archaeological evidence. Unlike traditional investigations of phonotactic constraints which focus on language-specific patterns, our study reveals a property valid across languages and writing systems. As both language and writing are unique aspects of our species, this universal signature may reflect an innate feature of the human cognitive phenomenon.

## Introduction

Language—and by extension, writing—distinguishes humans from all other species [1]. The ability to communicate complex information across both space and time have enabled society and civilization to emerge [2]. The recent availability of publicly accessible “big data”, such as the large digitized corpus on the *Google Books* website, has revolutionized the quantitative analysis of socio-cultural phenomena and led to new empirical discoveries [3–5]. Language in its written form is represented as symbolic sequences that convey information. Statistical analysis of such sequences have led to the identification of several quantitative properties that hold across many human languages. For example, one of the best known empirical regularities associated with language is the scaling behavior—referred to as Zipf’s law—that quantifies how some words occur far more frequently than others [6]. Several possible theoretical explanations of the phenomenon have been proposed [7, 8]. Words are themselves composed of signs corresponding to letters, syllabograms or logograms depending on the writing system. It has long been known that the different signs, e.g., letters, also occur with characteristic frequencies—a fact that has been used by cryptographers over the ages to break simple substitution ciphers. This was illustrated dramatically in fiction by Poe (*The Gold-Bug*) and Conan Doyle (*The Adventure of the Dancing Men*). For English, the phrase “ETAOIN SHRDLU” has often been used as a mnemonic for recalling the approximate order of the most commonly occurring letters in typical texts. However, a cursory glance through an English dictionary (or encyclopedia) to ascertain, for each letter of the alphabet, the number of pages that are required to list all the words (or entries) that begin with that letter, will alert one to a strong deviation from what is naively expected from the frequency distribution of letters. For instance, one of the letters having the largest number of entries in a dictionary is ‘c’ which does not even appear among the most frequently used letters in English as per the phrase above. This apparent anomaly arises from the fact that the letter ‘c’ has a much higher probability of occurring (relative to other letters) at the beginning of an English word—possibly a result of the specific orthography of English, where it can appear as the initial letter of the words *china*, *can*, *cent*, etc., in all of which it is pronounced differently—but does not occur so frequently at other positions. While it is rarer to come across situations where words are arranged according to their last character, it is possible to ask whether the frequency distribution of the letters that occur at the end of a word will similarly show a distinct character. For example, had we arranged the entries of an English dictionary in the order of the *last* letter of each word, we would see that this would result in the number of entries corresponding to each letter showing a far more unequal distribution than seen in a conventional dictionary. In other words, the last position in an English word is usually occupied by one of very few letters, suggesting that the final letter is much more tightly constrained than the initial letter. We show that this is not just true for English but holds in at least 23 other languages, including those which use writing systems not based on letters (alphabets and abjads), but instead on signs representing syllables or logograms. We have also applied this property of asymmetry in sign usage patterns between the beginning and end of a sequence to an undeciphered corpus of inscriptions, showing that we can determine the direction in which the sequences were written (left to right or right to left).

**Fig 1:**
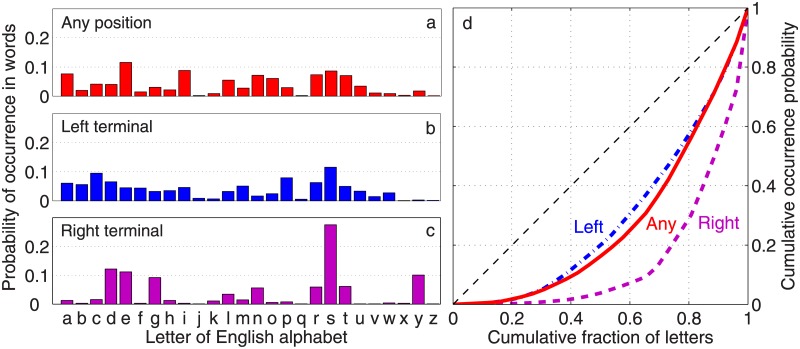
Unequal representation of letters (1-grams) occurring at different positions in words in written English. The probability of occurrence of the 26 letters of the English alphabet in the *Mieliestronk* corpus comprising about 58000 unique words of the English language (see Methods for details), at (a) any position, (b) left terminal position (i.e., in the beginning) and (c) right terminal position (i.e., at the end) of a word. The distribution shows more heterogeneity in the letter occurrence probabilities for (c), indicating that only a few letters occur with high frequency at the right terminal position of a word, compared to a relatively more egalitarian frequency of occurrence of letters in the left terminal position (b). This difference is illustrated in the Lorenz curve (d) comparing the cumulative distribution function for the occurrence probability of the different letters in any (solid curve), left terminal (dash-dotted curve) and right terminal position (dashed curve) of a word. The thin broken diagonal line (line of perfect equality) corresponds to a perfectly uniform distribution, deviation from which indicates the extent of heterogeneity of letter occurrence probability distributions—measured by the Gini index which is the ratio of the area between the line of perfect equality and the observed Lorenz curve, and, the area between the lines of perfect equality and of perfect inequality (viz., the horizontal line).

Fig 1(a)–1(c) shows the occurrence probability distribution of the 26 letters of the Latin alphabet at the initial and final positions of English words reconstructed from a large database, and compares it with their probabilities of occurring anywhere in a text. We note immediately that letters may differ greatly in terms of their occurrence probability depending on the position—but most importantly, the distribution for the right terminal character (the last letter) in an English word appears to be much more heterogeneous than the one corresponding to the left terminal character (the first letter). In other words, the choice of letters that can occur as the final character of a word is more restrictive, i.e., the occurrence probabilities are more unequal, with very few accounting for the right terminal position for a major fraction of the words, compared to their position-independent probabilities. In contrast, the probability distribution of letters that occur as the initial character is more egalitarian (in comparison to the final one), implying a somewhat higher degree of freedom of choice at the left terminal position. To ensure that this left-right asymmetry in sign usage distributions—suggesting a “handedness” of words in terms of the letter frequency distributions at their terminal positions—is not an artifact of the corpus one is using, we performed the same analysis with *Google Books Ngram* data, focusing on words that occur with a frequency of more than 10^5^ in the corpus digitized by Google (see Supporting Information for details). As seen from S1 Fig, the qualitative features are similar to that observed in Fig 1, indicating the robustness of the observed left-right asymmetry of sign occurrence probability patterns in words.

In this paper we will argue that this directional asymmetry is not just a feature of a particular language but appears to be universal, holding across different languages and writing systems. Regardless of whether the signs we are considering represent letters (for alphabetic scripts like English), syllabograms (for syllabic scripts such as Japanese Kana) or logograms (for logographic scripts like Chinese or logo-syllabic ones like Sumerian cuneiform), the distribution of the signs that begin a word shows relatively less heterogeneity than that for the ones that occur at its end. We have used measures of inequality (viz. Gini index and information entropy) to quantitatively assess the degree of asymmetry in the sign occurrence distributions for different linguistic corpora. The difference in the two distributions also indicate the differential information contents of the initial and final characters—and links our result to the statistical and information-theoretic analysis of language [9]. This approach was pioneered by Shannon who used the concept of predictability, i.e., the constraints imposed on a letter by those that have preceded it, to estimate the bounds for the entropy (the amount of information per letter) and redundancy in English [10, 11]. Considering the consequences of the most prominent structural patterns of texts—viz., the clustering of letters into words—Schürmann and Grassberger subsequently showed that the the average entropies of letters located inside a word are much smaller than that of the letters at the beginning [12, 13]. However, this is true even if one reverses the word—so that terminal letters of words (whether initial or final) have less predictability than those in other positions. Here we ask the relatively simpler question of whether the statistical properties of the left and right terminal characters are different and find a surprising non-trivial asymmetry in the heterogeneity of the respective distributions. Analysis of correlation between sign occurrences in written texts have traditionally focused on the phonotactic constraints of specific languages, e.g., determining the consonants or consonant clusters that are allowed to occur before and after a vowel in any syllable of a given language. While there is considerable variation between different languages as regards the possible arrangements in which consonants and vowels can be combined to make meaningful words, here we show the existence of general patterns that hold across many different language families.

## Results

In order to quantify the heterogeneity in sign usage distribution at the beginning and at the end of a word, we have used the Gini index or coefficient [14]. It measures dispersion in the distribution of a quantity and is widely used in the socio-economic literature to quantify the degree of inequality, e.g., in the distribution of income of individuals or households [15]. The value of the Gini index *G* (see Eq 1 in Materials and Methods) expresses the nature of the empirical distribution relative to a uniform distribution, with *G* = 0 if all values of the variable have the same probability of occurrence (“perfect equality”) while *G* = 1 corresponds to the extreme situation with the variable always taking up a single value (corresponding to a delta function probability distribution). Thus, if the probability of occurrence of any sign (e.g., the letters ‘A-Z’ in the case of the English alphabet) at the beginning (or end) of a word is about the same, the corresponding Gini index will be close to zero. Otherwise, it is a finite number (≤ 1) whose exact value depends on the extent of inequality in usage of the different signs. Measures related to the Gini index have previously had limited use in the context of linguistic sequences, e.g., to select attributes for decision tree induction in classification for data mining [16].

Using the Gini index on the distributions of letters (1-gram) that occur at the left and right terminal positions of words in English, we can quantitatively express the visible difference between patterns of unequal occurrence of signs at the two ends seen in Fig 1(b)–1(c). Fig 1(d) shows the Lorenz curve—a graphical representation of the inequality of a distribution—for the occurrence probability of the different 1-grams anywhere in a sequence as well as the two terminal positions. For a set of *N* symbols (*x*_1_, *x*_2_, … *x*_*N*_) that are indexed according to their probability of occurrence in non-decreasing order (*P*(*x*_*i*_) ≤ *P*(*x*_*i*+1_)), the curve is obtained by joining using linear segments the points (*X*_*i*_, *Y*_*i*_), *i* = 1, …*N*, where *X*_*i*_ = *i*/*N* is the cumulative proportion of the population of symbols and $Y_{i}=\Sigma_{j=1}^{i}P\left(x_{j}\right)/\Sigma_{q=1}^{N}P\left(x_{q}\right)$ is the cumulative proportion of occurrence probabilities. The diagonal line represents the case of complete equality, so that higher inequality is manifested as greater deviation between the empirical curve—showing the cumulative probability of occurrence of signs arranged in a non-decreasing order of occurrence frequency—and the diagonal. The diagram clearly shows that for different signs the probabilities of occurring at the right terminal position, i.e., the end of a word, is more unequal than their occurrence probability in the beginning (i.e., left terminal position of a word), or indeed, anywhere in a sequence. This quantitatively establishes that there is relatively more variation in the letters at the start of a word—and conversely less so when ending it. It indicates an inherent left-right asymmetry in the sign usage distribution of words in English that is related to the different degrees of freedom associated with choosing letters that begin and end a word. This asymmetry is more pronounced for letters or 1-grams, as measured by the normalized difference of Gini indices (defined as Δ*G* = 2(*G*_*L*_ − *G*_*R*_)/(*G*_*L*_ + *G*_*R*_) where *G*_*L*_ and *G*_*R*_ are the Gini indices for the signs occurring in the left and right terminal positions, respectively) for the two terminal positions, Δ*G* = −0.50 (with 95% bootstrap confidence intervals [−0.48, −0.51]; for details see Methods), than for 2-grams (Δ*G* = −0.25, 95% bootstrap confidence intervals [−0.247, −0.254]) and becomes even less noticeable for higher-order *n*-grams. We have therefore focused our analysis on using 1-grams for the subsequent results reported here.

To ensure that the left-right asymmetry does not arise simply as a result of the use of common prefixes (such as *de-* or *un-*) or suffixes (such as *-ed* or *-ly*) in English, we have also analyzed the Ogden list of Basic English words comprising two or more characters. This is a set of commonly used English root words obtained after removing all affixes or bound morphemes [17]. For this data-set, we obtain Δ*G* = −0.33 (with 95% bootstrap confidence intervals [−0.27, −0.47]) clearly indicating that the relative lack of variation in the right terminal position of a word in English is not an artifact resulting from, say, a large number of words ending with a limited set of suffixes.

**Fig 2:**
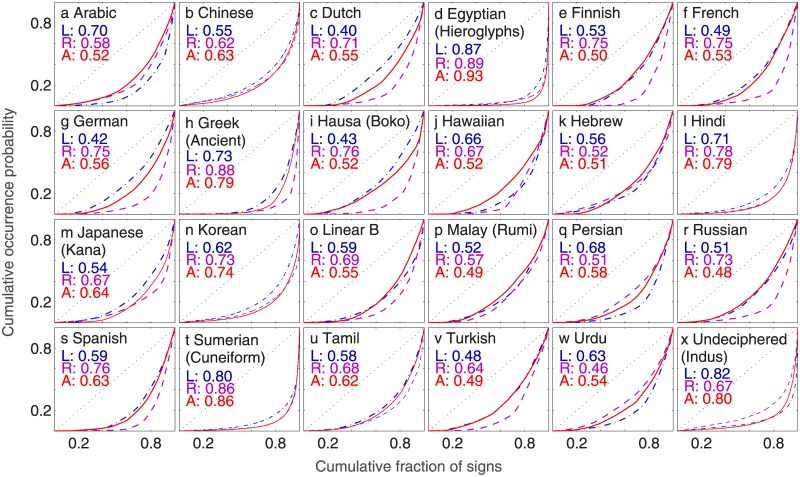
Unequal representation of signs (1-grams) occurring at different positions in words in corpora written using different languages and writing systems. The Lorenz curves in the 24 panels (corresponding to all the scripts analyzed here except English, which is shown in Fig 1) show the differences in the cumulative distribution function of the occurrence probability of signs at left terminal position (blue, dash-dot curve), right terminal position (purple, dashed curve) and at any position (red, solid curve) of a word written in a particular script. The thin broken diagonal line corresponds to a perfectly uniform distribution, deviation from which indicates the extent of heterogeneity of sign occurrence distributions. This is measured in terms of the Gini index, the corresponding values at the left terminal (L), right terminal (R) and any position (A) for a script being indicated in each panel.

**Fig 3:**
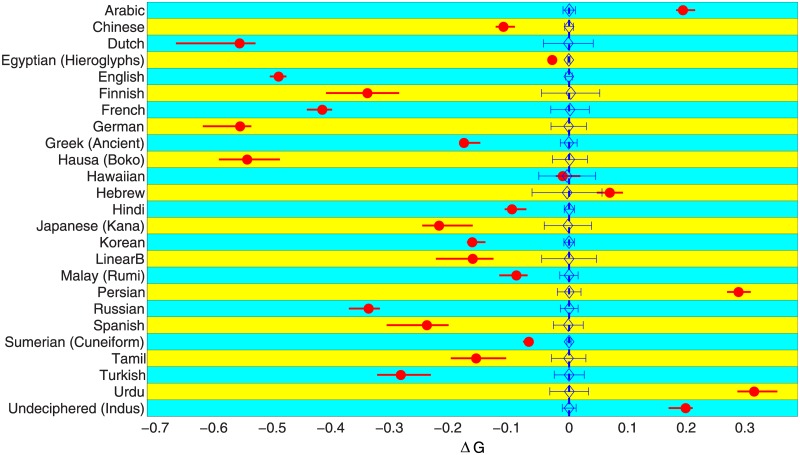
Asymmetry in the sign occurrence probability distributions at the left and right terminal positions of words in different languages correlate with the directions in which they are read. The normalized difference of the Gini indices Δ*G* = 2(*G*_*L*_ − *G*_*R*_)/(*G*_*L*_ + *G*_*R*_) (filled circles), which measures the relative heterogeneity between the occurrences of different signs in the terminal positions of words of a language, are shown for a number of different written languages (arranged in alphabetical order) that span a variety of possible writing systems—from alphabetic (e.g., English) and syllabic (e.g., Japanese kana) to logographic (Chinese) [see text for details]. All languages that are conventionally read from left to right (or rendered in that format in the databases used here) show a negative value for Δ*G*, while those read right to left exhibit positive values. The horizontal thick bars superposed on the circles represent the 95% bootstrap confidence interval for the estimated values of Δ*G*. To verify the significance of the empirical values, they are compared with corresponding Δ*G* (diamonds) calculated using an ensemble of 1000 randomized versions for each of the databases (obtained through multiple realizations of random permutations of the signs occurring in each word—see Materials and Methods for details), the ranges of fluctuations being indicated by error bars. Along with the set of known languages, Δ*G* measured for a corpus of undeciphered inscriptions from the Indus Valley Civilization (2600–1900 BCE) is also shown (bottom row).

While this asymmetry in the usage distribution for signs that begin and end words written in English is certainly striking, it would be even more significant if the phenomenon turned out to be valid for linguistic sequences in general. We have, therefore, carried out a systematic investigation of the inequality in sign usage distributions at the terminal positions of sequences that are chosen from languages spanning a broad array of language families. Fig 2 shows the Lorenz curves corresponding to these different corpora, each indicating the differences in the occurrence probabilities of signs at different positions for that language. The writing systems considered are also quite diverse, ranging from alphabetic to logographic, whose corresponding signaries (i.e., the set of distinct characters used for writing in that system) can vary in size from about two dozen to several thousands. Fig 3 shows the results obtained for the different written corpora we have analyzed, where the degree of asymmetry in sign occurrence at the left and right ends of a sequence is measured by the normalized difference Δ*G* between the respective Gini indices. The most important feature of our results is the clear distinction that can be made between languages that are conventionally written left to right, such as English, and those which are written right to left, such as Arabic, according to the sign of Δ*G* obtained for the corresponding corpus. A negative value of Δ*G* implies that the signs occurring in the left terminal position have a relatively more equitable distribution while the sign usage distribution at the right terminal position is more unequal, and conversely for positive Δ*G* comparatively few signs occur with high frequency at the left end of a sequence than the right end. Thus, our result implies that all languages and writing systems considered here exhibit an asymmetry between the beginning and end of a word in terms of the degree of inequality manifested in their respective sign occurrence probability distributions, the probability in choosing different signs being significantly more heterogeneous at the end than in the beginning.

To ensure that the observed distinction between the sign usage patterns at the two terminal positions of a sequence are significant, we compared our results with those obtained from corpora of randomized sequences, which by design have the same distribution of sign occurrences at all positions. For a rigorous comparison, we have used surrogate datasets that have the same frequency distribution of different signs as the original corpus (see Methods for details) so that any distinction between them arises only from differences in the nature of the distributions of sign occurrence at the terminal positions. As randomized sequences are expected not to have any left-right asymmetry in sign usage patterns, the mean value of Δ*G* for the surrogate data is expected to be zero. However, statistical fluctuations will result in the random corpora belonging to the ensemble having small non-zero values of Δ*G* distributed about 0 and the standard deviation of the distribution (indicated by error bars in Fig 3) indicates whether a observed difference in Gini indices can arise by chance even when there is no asymmetry. As seen in Fig 3 almost all the corpora analyzed by us exhibit asymmetries that are clearly distinct from what might be expected if they were just the result of noise.

**Fig 4:**
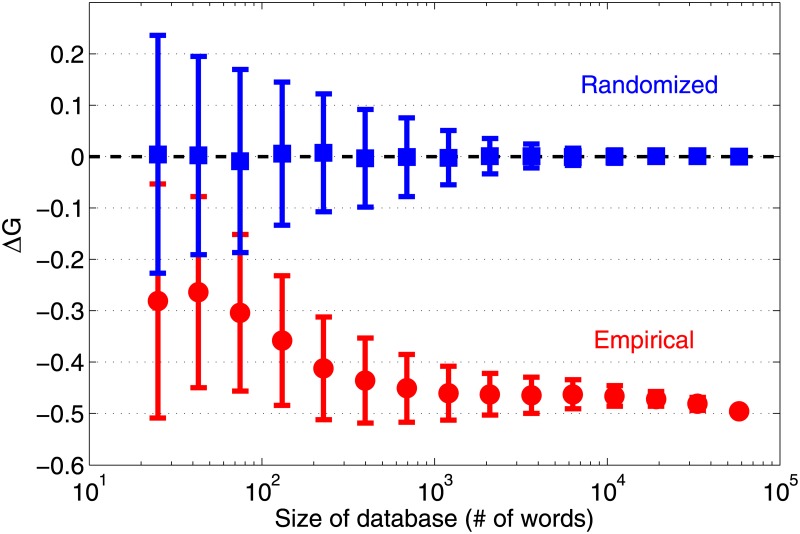
The observed asymmetry between heterogeneity of letter occurrence probability in left and right terminal positions is significant when the database is sufficiently large. Gini index differential Δ*G* shown for the left and right terminal letter (1-gram) distributions calculated using a set of *N* words, as a function of *N*. Empirical results are shown for random samples (without replacement) taken from the *Mieliestronk* corpus comprising about 58000 unique words of the English language, each data point (circles) being the average over 10^3^ samples of size *N*. For each empirical sample, a corresponding randomized sample is created by randomly permuting the letters in each of the *N* words, and a data point for the randomized set (squares) represents an average over randomizations of 10^3^ samples of size *N*. With increasing *N* the empirical distribution becomes distinguishable from the randomized set (which, by definition, should not have any left-right asymmetry). The error bars indicate standard deviation over the different samples.

As the asymmetry observed in the linguistic sequences should not depend on the particular corpus from which they are chosen, we obtained confidence intervals for the empirical Δ*G* values by bootstrap resampling of the data (see Methods for details). Fig 3 shows that in almost all cases this interval does not have any overlap with the interval obtained for randomized sequences—indicating that our results are robust with respect to variations in the corpus. The accuracy of the estimate, which is inversely related to the length of the confidence interval, appears to become higher as the database size, i.e., the total number of sequences being considered, is increased. Indeed, Fig 4 shows that the database needs to be larger than a minimal size (∼100 words for English) in order for the significance of the observed asymmetry to be established. Using increasingly larger databases, the difference between the empirical and randomized corpora become more pronounced.

Apart from the Gini index, the inequality in sign occurrence distribution at the terminal positions of a sequence can also be measured by other means, e.g., using information entropy (also referred to as Shannon entropy), a key concept in information theory. It measures the amount of uncertainty in a process by quantifying the non-uniformity in the probability distribution of different events. Thus, if all signs have equal probability of occurrence at a particular position in any sequence, this would correspond to the highest value of entropy (while the Gini index would have been the lowest in this case). On the other hand, if only a single sign occurs at this position in all sequences (i.e., the case of extreme inequality for which Gini index is highest), then the entropy is zero. S2 Fig shows that using the normalized difference of entropy Δ*S*, estimated from the sign occurrence distributions at the left and right ends of the different linguistic corpora, yields qualitatively similar results to those obtained by using the Gini index. The sign of Δ*S* in almost all cases is seen to be consistent with the direction of writing, with left-to-right written languages having positive values of Δ*S* (with the lone exception of Egyptian Hieroglyphics, for which we have used a database in which all sequences have been oriented so as to read from left to right for all our analysis) while those written right-to-left have Δ*S* < 0. This is in broad agreement with our earlier conclusion that there is relatively more equality in the probability of occurrence of different signs at the beginning of a word than at its end—reflected in the higher non-uniformity for sign usage distribution for the latter. Thus, it suggests that the asymmetry we observe in linguistic sequences may be robust with respect to the specific measure of inequality being used.

Intriguingly, we find that the asymmetry can also appear in a corpus of inscriptions that are so far undeciphered and whose relation to language is therefore not yet established. As an illustration, we have analyzed sign sequences appearing in the archaeological artifacts (e.g., seals, sealings, pottery, copper tablets, etc.) obtained from excavations carried out at sites of the Indus Valley Civilization (IVC) that existed during 2600–1900 BCE in present day Pakistan and northwestern India [18–20]. While there is some debate as to whether these inscriptions constitute “writing” in the sense of encoding spoken language [21, 22], there is near unanimity among scholars that these were mostly written from right to left as inferred from the archaeological evidence (e.g., signs get more crowded at the left end of some inscriptions or spill out of an otherwise linear arrangement) [23–26]. We have used a database where the relatively few sequences which are believed to have been written from left to right have been reversed so as to be oriented in the same direction as the majority, following standard procedure used for constructing concordances for Indus Valley Civilization inscriptions. We observe from Fig 3 that the Δ*G* for sign usage distribution is positive, indicating that the choice of signs is less restricted in the right terminal position than the left. This would suggest, based on the connection previously seen between the sign of Δ*G* and the direction of writing, that the IVC inscriptions are written from right-to-left, which corroborates the consensus view as mentioned above.

## Discussion

We have reported evidence here for a novel universal feature in the empirical statistics of linguistic sequences. Unlike the more well-known Zipf’s law and Heap’s law [27], which relate to the frequency of word usage, we focus on a more elementary level, viz., that of the signs—corresponding to letters, syllabograms or logograms, depending on the writing system—which constitute individual words. The distribution of occurrence for the different signs at the left and right terminal positions in a word are shown to have a distinct heterogeneous character that are characterized by measures of inequality such as the Gini index or information entropy. We observe that, in general, the information content at the beginning of a sequence tends to be higher than at the end, which is reflected in the significant asymmetry in terms of the restriction of sign usage at these the two positions. This is a pattern that is valid across different languages and scripts, possibly revealing a feature inherent in the information processing and communicating capabilities of the human cognitive apparatus.

The reason for the appearance of the directional asymmetry in sign usage distributions for linguistic sequences is yet to be definitively identified. However, it is not unreasonable to expect that this is related to the phonotactic constraints inherent in different languages. The initial sound of a word can be chosen with greater freedom from the set of all available speech sounds (phonemes) of the language, compared to all subsequent sounds that may depend—to a greater or lesser extent—on the sound(s) preceding them. For example, very few of the three-consonant clusters that can in principle occur in English are actually allowed [28]. Thus, one would expect a higher degree of variability in the initial sound compared to the one at the end of a word. As writing reflects the patterns of spoken language, to greater or lesser extent depending on the system, one would expect this difference between the beginning and end to be manifested in it. An indirect indication that phonotactic considerations may be at least partially responsible for the asymmetry is provided by the degree of the difference between the inequalities of sign usage at the two ends of a word in different writing systems—especially when normalized difference is used as a measure. We observe that, broadly speaking, the magnitude of Δ*S* (as well as, Δ*G*) is larger for scripts that have a higher proportion of phonetic representation [29, 30]. Thus, alphabetic and syllabic systems which have a much greater phonetic character than logographic or logo-syllabic systems tend to typically show a more pronounced asymmetry (S2 Fig). As even an apparently logographic system such as Chinese has some degree of phoneticism [29], it is not surprising that systems having a high degree of logography also show a difference in the sign usage distribution between the beginning and end of words, although this effect is much less marked than in other (more phonetic) scripts. There are exceptions to this general trend—for example, Hebrew, which is an alphabetic script, shows a low degree of asymmetry that is difficult to distinguish from effects due to stochastic fluctuations arising from sampling effects in a finite corpus. Hawaiian, which also appears to have a very low Δ*G*, however, shows a significant asymmetry when information entropy is used to measure sign usage inequality in place of the Gini index (see Supporting Information). Other indications that a simple phonotactic explanation for the observed asymmetry may not be adequate is shown by the fact that the relative position of some languages in terms of Δ*G* (or Δ*S*) do not necessarily conform to common perceptions about the degree of phonetic representation in the corresponding scripts used for writing them [30]. For example, the Korean han’gŭl script is considered to have a higher proportion of phoneticism than French [31]; however, the latter exhibits higher asymmetry in terms of both the measures of inequality used here (see Fig 3 and Supporting Information).

The asymmetry reported here may be used to infer the direction of writing, which is one of the basic pre-requisites for interpreting any linguistic sequence. A variety of possible directions have been seen in different writing systems, both historical and present [32]. The most common, left to right in horizontal lines, is the direction in which all scripts descending from the Greek and Brāhmī systems are written, including English, French, German, Hindi and Tamil. Scripts that are written in the other direction, i.e., right to left in horizontal lines, are also common and are used in ancient and modern Semitic scripts including Arabic and Hebrew. Another common orientation is from top to bottom in vertical columns, which is the direction in which Chinese and scripts influenced by it (such as Japanese) were traditionally written. Other, less common, directions of writing are also known, including bottom to top (the Celtic Ogham script) and *boustrophedon*, where the direction reverses in successive lines (as in archaic Greek and Luwian hieroglyph inscriptions). In cases where the inscriptions are undeciphered, such as those of IVC, the direction usually has to be inferred by indirect means. The asymmetry in sign usage patterns reported here—which shows that the beginning of sequences can be distinguished from the end by the nature of heterogeneity in the distributions of sign occurrence at these positions—can provide a valuable tool for ascertaining the direction of writing in such cases. Availability of a sufficiently large corpus would, however, be necessary for a reliable determination of the direction of writing in these inscriptions.

## Materials and methods

**Data description**. We have analyzed data from written corpora of twenty four languages (twenty two belonging to nine linguistic families, as well as, two language isolates), along with a corpus of undeciphered inscriptions from the Indus Valley Civilization (ca. 2600–1900 BCE). The writing systems considered range from alphabetic (that use only a few dozens of distinct letters) and syllabic to logo-syllabic and logographic (involving thousands of signs). The average corpus size is about ten thousand unique words collected from a variety of sources. Each word considered for our analysis consisted of multiple graphemes, corresponding to letters, logograms, hieroglyph signs or syllables depending on the writing system used. Detailed description of each corpus is provided in the Supporting Information.

**Estimation of occurrence probability distribution**. Probability distribution of sign occurrences in a corpus of inscriptions are estimated from frequency counts of the distinct signs appearing in the sequences belonging to the database, i.e.,

$$\begin{array}{c} \text{Prob}\left(\text{sign}\;i\;\text{in}\;\text{position}\;Q\right)=\frac{\text{Number}\;\text{of}\;\text{sequences}\;\text{where}\;\text{sign}\;i\;\text{occurs}\;\text{in}\;\text{position}\;Q}{\text{Total}\;\text{number}\;\text{of}\;\text{sequences}}. \end{array}$$

For establishing the directional asymmetry of sign usage, we focus specifically on the sign occurrence distributions at the left and right terminal positions of a sequence. The inequality of sign usage at these positions, which is reflected in the non-uniform nature of the corresponding distributions, is quantified by measuring the Gini coefficient or the information entropy.

**Measuring Gini coefficient**. The Gini coefficient or index is a measure of how unequal are the probabilities of all the different events that are possible. A value of zero for the coefficient corresponds to situations where all events are equally probable. Conversely, when only one event out of all possible ones is observed in every instance, the Gini coefficient attains its maximum value of 1. For a discrete probability distribution *P*(*x*), where the *N* possible values of the discrete variable *x* are indexed according to their probability of occurrence in non-decreasing order (*P*(*x*_*i*_) ≤ *P*(*x*_*i*+1_), *i* = 1, …, *N* − 1), the Gini coefficient [33] is measured as

$$\begin{array}{c} G=1-\left(1/N\right)\Sigma_{i=1}^{N}\left[P_{c}\left(x_{i}\right)+P_{c}\left(x_{i-1}\right)\right], \end{array}$$ (1)

where $P_{c}\left(x_{i}\right)=\Sigma_{j=1}^{i}P\left(x_{j}\right)$ is the cumulative probability of *x* with *P*_*c*_(*x*_0_) = 0 and *P*_*c*_(*x*_*N*_) = 1. For a given set of inscriptions, we estimate the cumulative probability distributions $P_{c}^{\left(L\right)}$ and $P_{c}^{\left(R\right)}$ for the signs *x*_*i*_ occurring in the left and right terminal positions, respectively, and use Eq 1 to compute the corresponding estimated Gini indices *G*_*L*_ and *G*_*R*_. The normalized difference between these two values,

$$\begin{array}{c} \Delta G=2\left(G_{L}-G_{R}\right)/\left(G_{L}+G_{R}\right), \end{array}$$

provides a measure for the asymmetry in the distribution of sign frequencies at the left and right terminal positions of the sequences.

**Estimating information entropy**. Apart from Gini index, we have used a measure based on information or Shannon entropy for quantifying the nature of the inequality of sign usage distributions at left and right terminal positions in a sequence. As entropy measures the unpredictability of information generated from a source, it can be used to characterize the underlying distribution of any process that produces a discrete sequence of symbols (chosen from a set of *N* possible ones) and is defined as

$$\begin{array}{c} S=-\Sigma_{i=1}^{N}P\left(x_{i}\right)\log_{2}\left(P\left(x_{i}\right)\right), \end{array}$$ (2)

where *P*(*x*_*i*_) is the probability of occurrence of the *i*-th symbol and the use of base 2 logarithm implies that the entropy can be expressed in units of bits [10]. In particular, given any database of inscriptions, we obtain estimates for the probabilities *P*^(*L*)^(*x*_*i*_) and *P*^(*R*)^(*x*_*i*_) for a particular sign *x*_*i*_ from the corresponding signary to occur in the left terminal and right terminal positions. After estimating these probabilities for all *N* signs that occur in the corpus of inscriptions, the left and right terminal entropies (*S*_*L*_ and *S*_*R*_, respectively) are calculated by using Eq 2. The normalized difference between the two entropy values, Δ*S* = 2(*S*_*L*_ − *S*_*R*_)/(*S*_*L*_ + *S*_*R*_), provides a measure of the degree of asymmetry in sign frequency at the two ends of a sequence.

**Bootstrap confidence intervals**. To quantify the degree of robustness in the estimates obtained using the empirical databases, we have used a bootstrap method to obtain confidence intervals for the measured values. For each corpus we have created 10^3^ resampled datasets (i.e., bootstrap samples) by random sampling with replacement, containing the same number of sequences as the original dataset. The probability distributions for sign usage at the terminal positions are then calculated for every bootstrap sample. Finally, a confidence interval for the normalized difference between the left and right terminal Gini indices, Δ*G* (or, of information entropy, Δ*S*) for the corpus is computed using the Bias-Corrected and accelerated (BCa) method which adjusts for bias in the bootstrap sample distributions relative to the actual sampling distribution [34]. The BCa confidence interval adjusts the percentiles of the bootstrap distribution of the parameter according to the calculation of a *bias correction* coefficient and an *acceleration* coefficient. The former adjusts for any skewness present in the bootstrap sampling distribution (it is zero if the distribution is symmetric). The latter coefficient adjusts for nonconstant variances (if any) in the resampled data.

**Sequence randomization**. The statistical significance of the measured asymmetry in sign usage is measured by comparing the results obtained from the empirical database with an ensemble of randomized surrogate sequence corpus. Each ensemble is generated by taking each sequence in turn that belongs to a database and doing a random permutation of the signs. This reordering ensures that the frequency distribution of the signs in each sequence (and thus, also the corpus) is unchanged in the randomized set, although all correlations (that contribute to 2- and higher order *n*-gram distributions) are disrupted. We then perform the same calculations as for the original empirical data for measuring the degree of asymmetric sign usage in left and right terminal positions of these randomized sequences—which, by design, are expected not to have any asymmetry. Significant difference in the results of the two datasets ensures that the measured asymmetry is not arising from stochastic fluctuations.

## Supporting information

S1 Fig Unequal representation of letters (1-grams) occurring at different positions in words in written English is robust with respect to choice of corpus. The probability of occurrence of the 26 letters of the English alphabet in the *Google Books Ngram* data comprising about 97000 unique words of the English language that occur with a frequency of more than 100,000 in the corpus (see Methods for details), at (a) any position, (b) left terminal position (i.e., in the beginning) and (c) right terminal position (i.e., at the end) of a word. While there are differences in the occurrence probability of the individual letters with the distribution shown in Fig 1 (see main text), as with the *Mieliestronk* corpus there is higher heterogeneity in (c) indicating that only a few letters occur with high frequency at the right terminal position of a word, compared to a relatively more egalitarian frequency of occurrence of letters in the left terminal position (b). This difference is illustrated in the Lorenz curve (d) comparing the cumulative distribution function for the occurrence probability of the different letters in any (solid curve), left terminal (dash-dotted curve) and right terminal position (dashed curve) of a word. The thin broken diagonal line corresponds to a perfectly uniform distribution, deviation from which indicates the extent of heterogeneity in the occurrence probabilities of different letters—measured as the ratio of the area between the line of perfect equality and the observed Lorenz curve, i.e., the Gini index.
(EPS)

S2 Fig Asymmetry in the sign occurrence probability distributions at the left and right terminal positions of words in different languages appear to be relatively robust with respect to the quantitative measure of inequality used. The normalized difference of the Shannon or information entropies Δ*S* = 2(*S*_*L*_ − *S*_*R*_)/(*S*_*L*_ + *S*_*R*_) (filled circles), which measures the relative heterogeneity between the occurrences of different signs in the terminal positions of words of a language, are shown for a number of different written languages (arranged in alphabetical order) that span a variety of possible writing systems—from alphabetic (e.g., English) and syllabic (e.g., Japanese kana) to logographic (Chinese) [see text for details]. Almost all languages that are conventionally read from left to right (or rendered in that format in the databases used here) show a positive value for Δ*S* (with the exception of Egyptian hieroglyphs), while those read right to left exhibit negative values. The horizontal thick bars superposed on the circles represent the bootstrap confidence interval for the estimated values of Δ*S*. To verify the significance of the empirical values, they are compared with corresponding Δ*S* (diamonds) calculated using an ensemble of randomized versions for each of the databases (obtained through multiple realizations of random permutations of the signs occurring in each word). Data points are averages over 1000 random realizations, the ranges of fluctuations being indicated by error bars. Along with the set of known languages, Δ*S* measured for a corpus of undeciphered inscriptions from the Indus Valley Civilization (2600–1900 BCE) is also shown (bottom row).
(EPS)

S3 Fig The observed symmetry between heterogeneity of letter occurrence probability in left and right terminal positions of words in a given language converges towards a value that is corpus size independent when the database is sufficiently large. Gini index differential Δ*G* shown for the left and right terminal letter (1-gram) distributions calculated using a set of *N* words, as a function of *N*. Empirical results are shown for random samples (without replacement) taken from a corpus of 10000 unique words in the Persian language, each data point (circles) being the average over 10^3^ samples of size *N*. For each empirical sample, a corresponding randomized sample is created by randomly permuting the letters in each of the *N* words, and a data point for the randomized set (squares) represents an average over randomizations of 10^3^ samples of size *N*. With increasing *N* the empirical distribution becomes distinguishable from the randomized set (which, by definition, should not have any left-right asymmetry) and converges to a value that is relatively independent of *N*. The error bars indicate standard deviation over the different samples.
(EPS)

S4 Fig Entropy of frequently occurring signs in the left terminal position of Egyptian hieroglyphic inscriptions are consistently higher than those occurring in the right terminal position. However, when one considers all signs that occur at the two ends, the entropy difference Δ*S* is marginally negative, inconsistent with the hypothesis that in writing systems read from left to right Δ*S* should be positive (in the Egyptian hieroglyph database used by us all sequences have been oriented so as to be read from left to right). The discrepancy is explained by the fact that many more rare signs (i.e., appearing with extremely low frequency) occur in the right terminal position than at the left terminal position. This is evident from the figure, where the curve corresponding to the right terminal position extends much further than the curve corresponding to the left terminal position. Note that if the entropy difference is calculated by considering the same number of signs at both ends, it is always positive which is consistent with the left-to-right orientation of the sequences according to the hypothesis presented here.
(EPS)

S5 Fig Variation of Gini index *G* as a function of sign position in words of a given length *L* (2 ≤ *L* ≤ 20) in written English. The inequality in the distribution of occurrence probabilities for letters of the English alphabet at different positions in words included in the *Mieliestronk* corpus shows *G* to be lower for the left-most sign compared to the right-most sign, consistent with the direction of the writing system. As English is read from left to right, the leftmost sign is numbered as 1, the one right to it as 2, etc. The second position (from left) appears to possess a higher value of *G* than either end of a word, suggesting that in English this is the most restrictive position in terms of the freedom in what signs can be used. The left panel shows the number of words of a given length that occurs in the corpus. Note that for longer words, the statistics is computed over very few exemplars. The length *L* of words is indicated along the vertical axis, while the horizontal axis shows the position *i* in a word of a given length.
(EPS)

S6 Fig Variation of Shannon entropy *S* as a function of sign position in words of a given length *L* (2 ≤ *L* ≤ 20) in written English. The inequality in the distribution of occurrence probabilities for letters of the English alphabet at different positions in words included in the *Mieliestronk* corpus shows *S* to be higher for the left-most sign compared to the right-most sign, consistent with the direction of the writing system. As English is read from left to right, the leftmost sign is numbered as 1, the one right to it as 2, etc. The second position (from left) appears to possess a lower value of *S* than either end of a word, suggesting that in English this is the most restrictive position in terms of the freedom in what signs can be used (consistent with results obtained by computing the Gini index *G* shown in S4 Fig). The left panel shows the number of words of a given length that occurs in the corpus. Note that for longer words, the statistics is computed over very few exemplars.
(EPS)

S7 Fig Variation of Gini index *G* as a function of sign position in words of a given length *L* (2 ≤ *L* ≤ 13) in written Persian. The inequality in the distribution of occurrence probabilities for letters of the Persian alphabet (a modified form of the consonantal Arabic alphabet) at different positions in words included in the Persian language corpus shows *G* to be lower for the right-most sign compared to the left-most sign, consistent with the direction of the writing system. As Persian is read from right to left, the rightmost sign is numbered as 1, the one left to it as 2, etc. No other position appears to have a characteristic trend in terms of sign usage inequality. The left panel shows the number of words of a given length that occurs in the corpus. Note that for longer words, the statistics is computed over very few exemplars.
(EPS)

S8 Fig Variation of Shannon entropy *S* as a function of sign position in words of a given length *L* (2 ≤ *L* ≤ 13) in written Persian. The inequality in the distribution of occurrence probabilities for letters of the Persian alphabet (a modified form of the consonantal Arabic alphabet) at different positions in words included in the Persian language corpus shows *S* to be higher for the right-most sign compared to the left-most sign, consistent with the direction of the writing system. As Persian is read from right to left, the rightmost sign is numbered as 1, the one left to it as 2, etc. No other position appears to have a characteristic trend in terms of sign usage inequality. The left panel shows the number of words of a given length that occurs in the corpus. Note that for longer words, the statistics is computed over very few exemplars.
(EPS)

S1 File Supporting information. Contains detailed description of the corpora, discussions on robustness of results and possible non-phonotactic mechanisms for emergence of asymmetry.
(PDF)

S2 File Supplementary data sets. Contains data-files (each in csv format) for the 25 different language and writing systems used in our study. The name of each data file mentions the corresponding language and a serial number (e.g., “01_Arabic.csv”) in accordance with the order in which the data sets are described in the Description of the Corpora in the Supplementary Material. Each sequence is represented as a string of numbers, with each number representing a specific grapheme for that language and writing system. In addition there are key lists for each of the data-bases (in txt format for the 24 known language and writing systems and in pdf format for the undeciphered Indus database) which shows the grapheme corresponding to each number that represents them in the data-base sequences. The name of each key list mentions the corresponding language.
(ZIP)
