# Supplementary material for: The “handedness” of language: Directional symmetry breaking of sign usage in words
Source: PLoS One. 2018 Jan 17;13(1):e0190735. doi: 10.1371/journal.pone.0190735 (PMC5771592; doi:10.1371/journal.pone.0190735)
Supplement: S1 File — Contains detailed description of the corpora, discussions on robustness of results and possible non-phonotactic mechanisms for emergence of asymmetry. (PDF) [file pone.0190735.s009.pdf]

Supporting Information for:

The “handedness” of language: Directional symmetry breaking of sign usage in words

Md. Izhar Ashraf[1,2], Sitabhra Sinha[1,3*],

1 The Institute of Mathematical Sciences, Chennai, Tamil Nadu, India.
2 B. S. Abdur Rahman University, Chennai, Tamil Nadu, India.
3 National Institute of Advanced Studies, Bengaluru, Karnataka, India.

These authors contributed equally to this work.
* sitabhra@imsc.res.in

Description of the corpora

Arabic: We have used a database of 14867 unique words (that are represented using two or more characters) of Classical (or Quranic) Arabic, a Semitic language written using a consonantal alphabet or ‘abjad’ [1]. The words are obtained from *Tanzil*, an international project started in 2007 to produce a standard Unicode text for the Qur’an (*http://tanzil.net/download/*, accessed: 25th March 2015). The signary comprises 36 signs, viz., 28 consonantal signs and 8 consonants with diacritical marks indicating vowels. The words range in length from 2 to 11 characters, the average length being 5.39.
Chinese: We have used a database of 13104 unique words (*ci*) of the Chinese language (belonging to the Sino-Tibetan language family) that are written using two or more signs (*zi*) [2] which have been obtained from a public-domain online *Chinese-English dictionary* CC-CEDICT (*http://www.mdbg .net/chindict/chindict.php?pahe=cc-cedict*, accessed: 8th January 2011). The database contains annotations identifying idiomatic expressions and loan words, and indicates proper nouns by capitalization of the corresponding English translation. From the total set of 115430 words and phrases available in the dictionary we have removed all single character words, idiomatic phrases, variants of the same word, hyphenated compound words, proper nouns and loan words. The Chinese writing system has been variously described as either logographic [1], or, an imperfect phonographic system with additional logographic attributes [3–5]. The signary for our data comprises 3691 distinct graphemes corresponding to logograms (hanzi). The sign sequences range in length from 2 to 9 signs, the average length being 2.46. Traditionally, Chinese is written top to bottom in vertical columns shifting from right to left; however, in modern times it is more frequently being written left to right in horizontal lines, and this is the convention used in our database. The words in our database are written using traditional Chinese characters.
Dutch: We have used a list of the 10000 most commonly used words in Dutch, a member of the Germanic branch of the Indo-European language family, from which we have chosen the 9146 unique non-hyphenated words comprising two or more characters. The data has been collected from the *Wortschatz* website maintained by the University of Leipzig (*http://wortschatz.uni-leipzig.de/Papers/top 10000nl.txt*, accessed: 22nd May 2015). The signary used has 31 distinct alphabetic characters

comprising 21 consonants, 5 vowels, 3 vowels with diacritical marks (acute accents or diaeresis), the digraph 'ij' that is considered as a letter in the Dutch language and an extra letter from the German alphabet (the *Eszett*). The words range in length from 2 to 25 letters, the average length being 7.6.
Egyptian (Hieroglyphs): Ancient Egyptian, a member of the Afro-Asiatic (Hamito-Semitic) language family, is written using a mixed system (also referred to as a logoconsonantal system [6]) with several hundreds of *hieroglyph* signs that can represent logograms, phonograms and/or determinatives [1]. We have used as data 39933 unique sequences comprising two or more hieroglyph signs of the *Middle Egyptian Dictionary* compiled by Mark Vygus (updated April 2015, *http://www.pyramidtext sonline.com/ MarkVygusDictionary.pdf*, accessed: 22nd May, 2015). The hieroglyphic signs are represented using the Gardiner sign list numbering system [7], the signary for the database used by us comprising 1859 distinct signs. The sign sequences range in length from 2 to 17 hieroglyphs, the average length being 5.12. The conventional direction of reading hieroglyphic sequences is "toward the face of human or animal pictograms, i.e., the signs are turned towards the beginning of the inscription" [6]. In the database used by us all sequences have been oriented so as to read from left to right.
English: We have used the *Mieliestronk* list of 58109 distinct words (comprising two or more letters) of the English language - belonging to the Germanic branch of the Indo-European language family - that has been compiled by merging several different word-lists (*http://www.mieliestronk.com/wordlist.html*, accessed: 4th December 2011). The words vary in length from 2 to 22 letters, the average being 8.34. The signary consists of the 26 lower case letters of the English alphabet. The list excludes spellings that are considered to be non-British. If a word is hyphenated, it is listed in unhyphenated form by removing the punctuation mark. The list contains some multiword phrases that are in common usage, rendered as a single word. Several words are included in both their singular and plural forms. Note that this is the word list for English that is included in the Supplementary Data Sets (S2 File).

For corroboration of our results obtained by analyzing the above dataset, we have also used statistics of sequence position-specific distributions of letter usage frequencies in a list of 97565 distinct words of the English language compiled from *Google books Ngram* data (English Version 20120701, in *http://storage.googleapis.com/ books/ngrams/books/datasetsv2.html*) by Peter Norvig and made freely available for public access (*http://norvig .com/tsv/ngrams-all.tsv.zip*, accessed: 22nd June 2015) [8]. Only those words are used which occur with a frequency of more than $100,000$ in the corpus of books scanned by Google. Details of the procedure used for compiling the N-gram frequency statistics are given in *http://norvig.com /mayzner.html* (accessed: 22nd June 2015). Note that, there are some differences in the relative frequency of occurrence of the different letters in the two databases. This is because, unlike in the preceding case where the occurrence probabilities are computed from a database comprising unique words, the Google 1-gram distributions are computed from corpora of books containing multiple occurrences of the same word. The frequencies of each letter is thus weighted by the occurrence frequencies in the corpus of different words (whose distribution follow Zipf's law) containing that letter. However, despite these differences in details, the inequality of sign usage at the right terminal positions is visibly higher than that in the left terminal positions (S1 Fig).

To ensure that our results are not an artifact of commonly used affixes (e.g., prefixes like *de-* or *un-* and suffixes like *-ed* or *-ly*) in English, we have also used the list of 850 words proposed by C. K. Ogden [9] as the core words of Basic English, a simplified subset of regular English. This word list is widely used as the beginner's vocabulary for the teaching of English as a second language. It comprises only word

roots, which for regular use are extended with different affixes. We have used the 848 words comprising two or more letters (i.e., omitting the single character words *a* and *i* from the Ogden list), which range in length between 2 and 14, the average length being 5.2.

In addition to these three data sets, we have tested several others of varying sizes including a lemmatised list of frequently used words from the British National Corpus [10], lemma being the canonical form of a word chosen to represent all the forms having the same meaning. All of these data sets exhibit similar left-right asymmetry as the above.

Finnish: We have used a list of the 10000 most commonly used words (all of which use two or more letters) in the Finnish language, belonging to the Finnic branch of the Uralic language family. The data, obtained from the *Wikiverb* website, has been collected from newsgroup discussions, press and modern literature (*http://wiki.verbix.com/Documents/WordfrequencyFi*, accessed: 24th June 2015). The signary used has 25 distinct signs - i.e., all vowels and consonants of the modern Latin alphabet excepting "q","x" and "w", along with two additional vowels "ä" and "ö". The words vary in length from 2 to 25 letters, the average being 7.81.

French: We have used a list of the 10000 most commonly used words in French, a Romance language belonging to the Indo-European family, from which we have chosen the 9189 unique words that have two or more characters. The data has been collected from the *Wortschatz* website maintained by the University of Leipzig (*http://wortschatz.uni-leipzig.de/Papers/top10000fr.txt*, accessed: May 22nd 2015). The signary used has 30 distinct alphabetic characters comprising 26 letters of the Latin alphabet along with 3 vowels with diacritical marks (acute accents or diaeresis) and an apostrophe sign. The words range in length from 2 to 19 letters, the average length being 7.7.

German: We have used a list of the 9172 most commonly used words in German, a member of the Germanic branch of the Indo-European language family, from which we have chosen the 9152 distinct words that have two or more characters. The data has been collected from the *Wortschatz* website maintained by the University of Leipzig (*http://wortschatz.uni-leipzig.de/ Papers/top10000de.txt*, accessed: May 22nd 2015). The signary used has 32 distinct alphabetic characters comprising the 26 letters of the Latin alphabet along with 4 vowels having diacritical marks (umlauts or acute accents), a ligature (the *Eszett* or *scharfes S*) and an apostrophe sign. The words vary in length between 2 to 27 letters, the average being 8.1.

Greek: We have used a list of the 10000 most frequently occurring words - grouped by lemma - in classical Greek literature written in ancient Greek which belongs to the Indo-European language family, compiled by Kyle Johnson from the *Thesaurus Linguae Graecae* corpus (maintained by University of California, Irvine) using the *Classical Language* Toolkit (*http://cltk.org*) and made freely available to the public (*http://kyle-p-johnson.com/assets/most-common-greek-words.txt*, accessed: 24th June 2015). From this dataset we have used the 9868 distinct words that have two or more characters. The signary has 124 distinct characters as the words are represented in the traditional polytonic orthography used for ancient Greek, involving 24 basic letters used in conjunction with several varieties of diacritical marks (e.g., accents, breathing marks, iota subscript and diaeresis). The words range in length from 2 to 18 characters, the average being 6.9.

Hausa (Boko): Hausa, a Chadic language belonging to the Afro-Asiatic family, is written using Boko, a Latin-based alphabet, which was devised in the 19th century and became the official system in the early part of the 20th century (in earlier periods, it was written in Ajami, an Arabic alphabet). We have used a list of 7062 unique words that have two or more characters obtained from a Hausa online dictionary

maintained by the University of Vienna (*http://www.univie.ac.at/Hausa/KamusTDC/CD-ROMHausa/KamusTDC/ARBEIT2.txt*, accessed: 19th May, 2015). The signary used has 30 distinct alphabetic characters comprising 23 letters from the Latin alphabet, four additional signs representing glottalized consonants, two digraphs ('sh' and 'ts') and an apostrophe sign. The words range in length from 2 to 22 characters, the average being 6.0.

Hawaiian: Hawaiian is Polynesian language belonging to the Austronesian family that had no written form until the 19th century when foreign missionaries devised an alphabetic system for recording it based on the Latin script. The data used for our analysis has been collected from the entries of *A dictionary of the Hawaiian language* (1922) compiled by Lorrin Andrews and revised by Henry H Parker (Board of Commissioners of Public Archives of the Territory of Hawaii, Honolulu) and freely available online (*http://ulukau.org/elib/cgi-bin/library?c=parker&l=en*, accessed on 28th May 2015). After removing all non-native words that contain characters that do not belong to the Hawaiian alphabet, we have compiled a data-base of 14009 unique words containing two or more characters. The signary comprises 28 distinct characters, with 12 basic letters - representing 5 vowels and 7 consonants - of the Hawaiian alphabet along with vowels used in conjunction with diacritical marks (breve and macron) indicating short or long pronunciation, and a sign to indicate glottal stop (the '*okina*). The words range in length from 2 to 26 characters, the average being 9.0.

Hebrew: We have used a list of the 10000 most commonly used words (compiled from online written texts) in modern Hebrew, a Semitic language written using a consonantal alphabet or 'abjad', from which we have chosen the 9993 distinct words that are represented using two or more characters. The data has been collected from a list maintained by *Teach Me Hebrew*, an online Hebrew language learning site (*http://www.teachmehebrew.com/hebrew-frequency-list.html*, accessed: 26th December 2013). The signary comprises 31 signs, viz., 27 consonantal signs (comprising 22 letters of which five use different forms - called *sofit* - when used at the end of a word) and 4 signs used in conjunction with *niqqud* diacritical marks. The words range in length from 2 to 13 characters, the average length being 5.1.

Hindi: Hindi is an Indo-Aryan language, a branch of the Indo-European family, which is written in the Devanagari script that is sometimes classified as an alphasyllabary [6] or 'abugida' [11]. Like the other writing systems that are descended from the Brāhmī script of ancient India, Devanagari uses as its main functional unit the *aksara*, which may consist of only a vowel but more frequently represents a syllable consisting of a consonant and an inherent vowel along with diacritical marks that may indicate use of other vowels [1]. We have used a database of 6441 distinct words written using two or more characters, collected from an online dictionary (*Shabdanjali*) of Hindi developed by the Language Technology Research Center at Indian Institute of Information Technology, Hyderabad (*http://ltrc.iiit.ac.in/showfile.php?filename=downloads/shabdanjali-stardict/index.html*, accessed: 20th May 2015). The signary comprises 571 distinct signs, comprising 11 vowels, 33 consonants, their conjunctions with each other and with consonantal sound modifiers (the *anusvara*, *chandrabindu*, *visarga* and *halant*). The words range in length from 2 to 10 signs, the average length being 3.5.

Japanese (Kana): Japanese, which belongs to the Japonic language family, is written using a combination of the logographic *Kanji* system (adopted from Chinese characters) and the syllabic *kana* system. The latter, in turn, consists of a pair of distinct syllabaries: *hiragana*, used for writing native Japanese words and *katakana*, which is used for foreign words. For our study, we have focused only on the syllabic writing system for Japanese. We have used a list of 1162 distinct words written using two or more signs from the kana syllabary, which is obtained from a list of common Japanese words collected from textbooks used by foreign learners of the language and

maintained by *Japanese Words*, an online site for learning the Japanese language (*http://www.japanesewords.net/36/over-1000-japanese-words-list/*, accessed: 29th May 2015). The signary has 103 distinct characters comprising 46 basic signs of Hiragana and 21 basic signs of Katakana, 22 Hiragana and 9 Katakana signs used in conjunction with diacritical marks (the *dakuten* and *handakuten*), smaller forms of 4 hiragana characters (viz., of *ya*, *yu* and *yu* which indicate the yōon feature, and the *sokuon* used to mark a geminate consonant) and a special symbol (*chōonpu*, the long vowel mark). The words range in length from 2 to 13 characters, the average being 3.8.

Korean: Korean is a language isolate with no established connection to any of the major language families of the world and is written using Han'gŭl, a purely phonetic script, although in earlier times a system based on Chinese characters (Hanja) was used. Each character corresponds to a syllable, the syllabic block being composed of two to six letters (including at least one consonant and one vowel) from the basic alphabet comprising 10 vowels, 14 consonants and 27 digraphs. The number of possible distinct syllabic blocks or characters exceeds 11,000 although a far smaller number is in actual use [1]. We have used a list of 5888 commonly used words compiled by the National Institute of Korean Language in 2004 (publicly accessible from *https://en.wiktionary.org/wiki/Wiktionary:Frequency_lists/ Korean_5800*, accessed: 24th June 2015) from which we have chosen 5141 distinct words that are represented using multiple syllabic blocks. The signary comprises 966 distinct characters (each corresponding to a syllabic block). The words range in length from 2 to 6 characters, the average length being 2.7.

Linear B: Linear B is a syllabic script, with most of its signs representing consonant-vowel combinations, that was used for writing archaic Greek between 1500 and 1200 BCE. We have used as data 1924 distinct sequences comprising two or more characters from the *Linear B Lexicon* compiled by Chris Tselentis (*https://www.scribd.com/doc/56265843/ Linear-B-Lexicon*, accessed: 15th May 2015). The signary comprises 87 distinct signs representing syllables. The words range in length from 2 to 8 signs, the average being 3.8.

Malay (Rumi): We have used a list of the 10000 most commonly used words in Malay, a member of the Austronesian language family, from which we have chosen the 9970 unique words that have two or more characters. All words are written in *Rumi* or Latin script, which is the most commonly used form for writing Malay at present, although a modified Arabic script (*Jawi*) also exists. The data has been collected from the list of high frequency words that are publicly available at *Invoke IT Blog* (*https://invokeit.wordpress.com/frequency-word-lists/*, accessed: 4th January, 2014). The signary comprises the 26 letters of the Latin alphabet. The words range in length from 2 to 17 letters, the average being 6.8.

Persian: We have used a list of 10000 most commonly used words (each represented using two or more characters) in Persian, a member of the Indo-Iranian branch of the Indo-European language family, which is written using a modified form of the consonantal Arabic alphabet or 'abjad'. The words are obtained from a list of high-frequency words compiled using the Tehran University for Persian Language corpus and available at *Invoke IT Blog* (*https://invokeit .wordpress.com/frequency-word-lists/*, accessed: 4th January 2014). The signary comprises 40 signs, viz., 32 consonantal signs, a long vowel indicator ('alef madde'), a ligature ('lām alef'), a diacritic ('hamze'), 3 consonants with the 'hamze' diacritical mark and different forms for the consonants 'kâf' and 'ye' when they occur in final position. The words range in length from 2 to 13 letters, the average being 5.2.

Russian: We have used a list of 9011 distinct words that use two or more characters in Russian, a member of the Slavic branch of the Indo-European language family and which is written using a Cyrillic alphabet. The data has been collected from *Russian*

Learners' Dictionary: 10,000 words in frequency order compiled by Nicholas J Brown (Routledge, London, 1996), after removing all words that use characters not in the standard Russian alphabet. The signary comprises the 33 letters of the modern Russian alphabet. The words range in length from 2 to 21 letters, the average being 8.0.

Spanish: We have used a list of 4902 distinct high-frequency words (that use two or more characters) in Spanish, a Romance language belonging to the Indo-European family. The data has been collected from *A Frequency Dictionary of Spanish* compiled by Mark Davies (Routledge, London, 2006). The signary used has 35 distinct alphabetic characters comprising 26 letters of the basic Latin alphabet along with an additional character ñ and two digraphs ('ch' and 'll'), as well as, vowels with diacritical marks (acute accents or diaeresis). The words range in length from 2 to 19 letters, the average being 7.4.

Tamil: Tamil is a Dravidian language is written in a script (sometimes classified as an 'abugida') derived from Brāhmī script and thus shares a common origin with the Devanagari script used for writing Hindi (see above) although it differs significantly both in appearance and structure [1]. It has 31 basic signs consisting of 12 vowels, 18 consonants and a special character, with combinations of the different vowels and consonants yielding a possible 216 compound letters. Additional characters from the Grantha script and diacritical marks are sometimes used to represent sounds not native to Tamil, e.g., in words borrowed from other languages. As with other Indian scripts, Tamil uses the *aksara* as its basic unit - however, unlike then it has eliminated most conjuncts, consonant clusters being placed in a linear string. The data used for our analysis has been collected from texts (e.g., Paripaadal, Thiruppavai, Kamba Ramayanam, Sundara Kandam, Akananooru songs, etc.) available in *Chennai Library*, an online repository of Tamil literature (*http://www.chennailibrary.com*, accessed: 27th December 2012). From this a data-base of 1991 unique words containing two or more characters was compiled. The signary comprises 187 distinct signs corresponding to different basic and compound letters and the special character. The words range in length from 2 to 9 letters, the average being 3.8.

Turkish: We have used a list of 9909 distinct high-frequency words (that use two or more characters) in Turkish, a member of the Turkic language family. The data has been collected from a *Wiktionary* word frequency list (*https://en.wiktionary.org/wiki/ Wiktionary:Frequency_lists/ Turkish_WordList_10K*, accessed: 14th July 2015). The signary used has 32 letters, comprising 29 letters of the Turkish alphabet and 3 vowels used in conjunction with circumflex accents. The words range in length from 2 to 17 letters, the average being 6.9.

Sumerian (Cuneiform): Sumerian, a language isolate spoken in ancient Mesopotamia during the 3rd millennium BCE, was written using a logosyllabic system with several hundred signs of a cuneiform script representing logograms, phonograms and/or determinatives. We have used as data 19221 unique words (comprising two or more cuneiform signs) collected from texts available in the *Electronic Text Corpus of Sumerian Literature* (ETCSL, *http://etcsl.orinst.ox.ac.uk*, accessed: 20th October 2010). The signary comprises 1364 distinct transliteration values of Cuneiform signs that represent syllables. The sequences range in length from 2 to 11 signs, with the average being 3.6.

Urdu: We have used a database of 4998 unique words that are represented using two or more characters in Urdu, an Indo-Aryan language belonging to the Indo-European family, that is written using an extended Persian alphabet. The words are obtained from a list of frequently used words maintained by the Center for Language Engineering at Lahore (*http://www.cle.org.pk /software/ling_resources/UrduHighFreqWords.htm*, accessed: 1st January 2014). The

signary comprises 46 signs, viz., 38 consonantal signs, 3 long vowels ('alef madde', 'lām alef madde' and 'ya'), 2 semi-consonants ('hamzah' used in conjunction with 'wao' or 'ya'), a nasalized consonant ('noon ghunna'), a ligature ('lām alef') and an additional sign ('ta' marbuta') used for writing certain loan-words. The words range in length from 2 to 11 letters, the average being 4.6.

Undeciphered (Indus): As an example of an undeciphered corpus on which to apply our analysis, we have used the set of inscriptions obtained from archaeological excavations at various sites of the Indus Valley civilization (ca. 2600-1900 BCE). The data used for our analysis is collected from the ICIT Database of Indus Writing compiled by Bryan K. Wells [12, 13] and maintained by Andreas Fuls (*http://caddy.igg.tu-berlin.de/indus/welcome.htm*, accessed: 20th October 2017) from which we have removed all incomplete and multiple-line inscriptions thereby obtaining 1837 unique sequences that contain two or more signs. The Indus signs are represented using the Wells sign list numbering system [12, 13], the signary for the database used by us comprising 568 distinct signs. The sequences range in length from 2 to 13 signs, the average being 4.6. The direction of the sign sequences vary, the majority being written right to left, although examples of left to right also exist, as inferred from external evidence such as signs becoming relatively cramped towards the end of a sequence inscribed on an archaeological artifact (e.g., seals, tablets or potsherds) [14, 15]. In the database used by us [13], the relatively few sequences which are believed to have been written from left to right have been reversed so as to be oriented in the same direction as the majority (i.e., which are believed to be written from right to left). This follows the standard procedure used also for constructing earlier concordances for Indus Valley Civilization inscriptions [14, 15]. Note that if only the 1779 sequences which are believed to be written from right to left are considered for our analysis, we obtain $\Delta G = 0.20$ and $\Delta S = -0.41$ which are identical to the values reported in the main text for the database in which all 1837 sequences are oriented right-to-left.

Robustness of results

An important consideration when quantifying the inequality of sign usage is the size of the signary, i.e., the number of visually distinct signs (sometimes referred to as 'graphs' [16]) that can be identified in each corpus. In several scripts, complex characters that are recognizably the compound of two or more basic characters are quite commonly used - as in the system of conjunct consonant signs or ligatures in the Devanagari script used for writing Hindi and other South Asian languages - and in principle, one could either consider these as separate signs or decompose them into the constituent signs, which will result in very different signary sizes. Also, Semitic scripts such as Arabic and Hebrew that are essentially consonantal alphabets often use diacritical marks for indicating vowel usage. In such cases, the same consonant is used in conjunction with different diacritics when the vowel following it is different. The signary size would depend upon whether these are considered to be distinct signs or not. In several other scripts, special marks can be used together with the vowels and consonants, e.g., the use of apostrophe to indicate the omission of one or more sounds in European languages such as French or German, and the use of a glottal stop marker (*'okina*) in Hawaiian. Once again, whether these signs are treated as distinct elements or part of the associated letter will affect the signary size. We observe that although the numerical values of the Gini indices (and information entropy) can be affected by changes in the signary size, the asymmetry in terminal sign usage reported here is robust with respect to these choices about different conventions for identifying distinct signs constituting the signary for a given corpus.

We have also examined our results for their dependence on corpus size, i.e., the number of words included in each database. As shown in Fig 4 in the main text (for English) and S3 Fig in Supplementary Information (for Persian), the scores for each language indicative of their asymmetry converge to a value which is relatively independent of the corpus size as the number of words included in the corresponding database is increased. We have explicitly checked that the scores for the different languages attain asymptotic values (for large enough database sizes) which are distinct from each other. Thus the variation of the scores for the different languages cannot be attributed to corpus size alone.

Possible non-phonotactic mechanisms for emergence of asymmetry

It is possible that the asymmetry we have reported here could arise in certain situations for reasons other than phonotactic constraints. For instance, in the undeciphered IVC inscriptions, the most frequently occurring sign - viz., the U-shaped "jar" symbol [17] - that appears very frequently at the end of a sequence, dominates the probability distribution of signs that can occur at the left terminal position (accounting for about a third of all the distinct sequences comprising the corpus). By contrast, the most frequently occurring sign at the right terminal position is seen to begin only about 6 % of the sequences. These distinctive sign usage patterns at the two terminal positions of IVC sequences gives rise to the heterogeneity in the corresponding occurrence probability distributions. In the absence of a decipherment, it is purely speculative whether the inequality arises for phonotactic reasons (as in the linguistic sequences considered here) or a fortuitous stylistic convention.

Appearance of sign usage asymmetry in writing systems that are not alphabetic or syllabic

One of the writing systems included in our study, that of Chinese language, is traditionally considered to be logographic [1]. It is thus worth considering whether a non-phonotactic mechanism could be responsible for the appearance of sign usage asymmetry in such a system. It has been reported that, while single signs or characters (*zì*) can indeed represent words, the majority of Chinese words (*cì*) are written using multiple signs, some studies suggesting as much as 70% of commonly used words involve two or more signs [18]. While some of these words are polysyllabic, several others are compound words created by affixation, i.e., combining a word with a prefix, infix or suffix. As usage of suffix in Chinese is much more common than other types of affix usage [19], this may possibly be a mechanism by which sign usage asymmetry can arise (e.g., the use of a limited set of suffixes at the end of a multi-character word can reduce the diversity of sign usage at that position). However, we have carried out analysis of the database of Chinese words after eliminating words with affixes and observed that the sign usage asymmetry still persists. A plausible hypothesis one may consider is that the remaining words in the database possess some degree of phonetic character. Thus, some variant of the phonotactic argument for the observed asymmetry suggested in the main text may arise even for this system. We note that several authors have indeed argued that, instead of treating it as a purely logographic system [1], Chinese writing is better thought of as an "imperfect phonographic system with additional logographic attributes" [3]. Indeed the importance of phonetic information in the development of the Chinese writing system

is considered to be unequivocal [5].

We also note that other writing systems that use logograms, such as the Egyptian hieroglyphic system, are nevertheless phonological in character to a considerable degree. Indeed, the recognition that Egyptian hieroglyphic writing system is significantly phonetic was crucial to its decipherment by Jean-Francois Champollion [20]. Egyptian represents an apparently unique polyconsonantal system [5], with extensive use of determinatives that appear at the end of words (following the phonetic signs) supplying specified meanings. It is intriguing to consider whether a variant of the phonological argument that has been suggested to underlie the sign usage asymmetry in alphabetic and syllabic systems could also apply to these apparently logographic and logoconsonantal systems.

Entropy difference in Egyptian hieroglyphs

As can be seen from S2 Fig, for almost all sequence databases that are read from left to right (or rendered in that format) we see a positive value of ΔS except for Egyptian hieroglyphs. Instead of having a higher entropy for the left terminal sign compared to right terminal sign (as should have been the case given that all sequences have been oriented to be read from left to right in the database), it exhibits a marginally higher entropy for right terminal sign in comparison to left terminal sign. Note that the numerical value of ΔS for this data-set lies almost within the error bars of the corresponding randomized data-set, so that the direction cannot be conclusively determined given the data. The discrepancy may also be connected to a distinctive feature of Egyptian hieroglyphic inscriptions, viz., there are many more rare or low-frequency signs that can appear in the right terminal position but never in the left terminal position. Thus, there are 1502 distinct signs that appear in the right terminal position of a sequence while only 798 distinct signs appear in the left terminal position. If we compute the entropy of the characters occurring in the left and right terminal positions by confining our attention to the r most frequently occurring signs for either position (so that the number of terms used for computing the two entropy values are comparable) we note that the left terminal sign entropy is consistently higher than the right terminal sign entropy for all r (see S4 Fig). This is consistent with our hypothesis according to which the left terminal position should have exhibited higher entropy.

Inequality of sign usage distributions at different positions in a sequence

In the main text we have only considered the difference in the occurrence probability of signs at the two extreme positions of a sequence, viz., at its beginning and at its end. However, one can also consider the sign usage distribution at other positions in a sequence, and in particular, ask whether there is a systematic variation in the inequality measures of sign usage frequencies with the relative position of their occurrence in a sequence. To study this, instead of considering the entire data-set of sequences in a given language (e.g., English words) together, we split it into sub-sets, each having sequences of the same length. This is necessary as otherwise, it is difficult to compare the properties of (say) the 4th character in a sequence when the sequence itself is a 4-letter word (in which case, the character is also the right terminal symbol) and the 4th character in a 7-letter world (in which case it is in the middle of the sequence).

We show in S5 and S6 Figs the variation of the Gini index G and the entropy S (respectively) as a function of sign position in words of a given length L, where L

varies from 2 to 20. Note that the number of words of different lengths vary considerably in the data-set as shown in the side-panel in the left (showing the frequency of occurrence of words of a given length in the *Mieliestronk* English corpus data-base described above). Thus the variation in G (or S) with position in the much longer words are based on statistics computed over extremely few exemplars. However, broadly we can see that the Gini index for the left-most sign is lower than that for the right-most sign in English words supporting our hypothesis. Note that, it appears that the second position from left appears to have a much higher G value than either its left or right neighbour. However, this is unique to the English database and such a feature cannot be seen in other language data-bases that we have analyzed.

When we look at the variation of S with sign position for words of a given length, we find that the entropy of the left-most sign is higher than the right-most sign in English words, which is again consistent with our hypothesis. The other positions do not exhibit any consistent variation except for the second sign starting from the left which (consistent with what was seen for G) has an entropy value that is lower than its left or right neighbour. Again, this seems to be an unique feature of the English database as we do not see it occur in the data-bases for other languages.

For example, in S7 and S8 Figs we show G and S, respectively, as a function of sign position in words of a given length L for the Persian language database. As Persian is read from right to left we have numbered the rightmost sign as 1, the one left to it 2 and so on. Consistent with our hypothesis we find that G for the left-most sign is higher than that for the right-most sign and the S for the left-most sign is lower than that for the right-most sign. However, no other sign appears to exhibit any characteristic trend in the variation of either G or S. One may therefore conclude that a predictable difference in G (and S) for the signs occurring in the left-most and right-most positions of sequences in a given language is possibly the only consistent feature seen across the languages and writing systems considered by us.

References

1. Coulmas F. Writing Systems: An Introduction to their Linguistic Analysis. Cambridge, UK: Cambridge University Press; 2003.

2. Chao YR. Language and Symbolic Systems. Cambridge, UK: Cambridge University Press ;1968.

3. DeFrancis J. The Chinese Language. Honolulu: University of Hawaii Press; 1984.

4. DeFrancis J. Visible Speech: The Diverse oneness of writing systems. Honolulu: University of Hawaii Press; 1989.

5. Sproat R. A Computational Theory of Writing Systems. Cambridge, UK: Cambridge University Press; 2000.

6. Daniels PT, Bright W, editors. The World's Writing Systems. New York: Oxford University Press; 1996.

7. Gardiner A. Egyptian Grammar: Being an Introduction to the Study of Hieroglyphs. 3rd ed. Oxford: Griffith Institute; 1957.

8. Norvig P. English letter frequency counts: Mayzner Revisited or ETAOIN SRHLDCU; 2013. Available from: http://norvig.com/mayzner.html Cited 22 June 2013.

9. Ogden CK. Basic English: A General Introduction with Rules and Grammar. London: Paul Treber; 1930. Word list available from: http://ogden.basic-english.org/ Cited 31 May 2016.

10. Kilgarriff A. Putting frequencies in the dictionary. International Journal of Lexicography. 1997; 10: 135-155.

11. Daniels PT. Fundamentals of grammatology. Journal of the American Oriental Society. 1990; 100: 727-731.

12. Sinha S, Ashraf MI, Pan RK, Wells BK. Network analysis of a corpus of undeciphered Indus civilization inscriptions indicates syntactic organization. Computer Speech and Language. 2011; 25: 639-654.

13. Wells BK. The Archaeology and Epigraphy of Indus Writing. Oxford: Archaeopress; 2015.

14. Mahadevan I. The Indus Script: Texts, Concordance and Tables. Calcutta: Archaeological Survey of India; 1977.

15. Parpola A. Deciphering the Indus script. Cambridge, UK: Cambridge University Press; 1994.

16. Coulmas F. The Blackwell Encyclopedia of Writing Systems. Malden: Blackwell; 1996.

17. Possehl G. The Indus Civilization: A Contemporary Perspective. Lanham: AltaMira Press; 2002.

18. Yip PC. The Chinese Lexicon: A Comprehensive Survey. New York: Routledge; 2000.

19. Li CN, Thompson SA. Mandarin Chinese: A Functional Reference Grammar. Berkeley: University of California Press; 1981.

20. Bard KA. An Introduction to the Archaeology of Ancient Egypt. Chichester: John Wiley; 2015.
