# Supplementary material for: The “handedness” of language: Directional symmetry breaking of sign usage in words
Source: PLoS One. 2018 Jan 17;13(1):e0190735. doi: 10.1371/journal.pone.0190735 (PMC5771592; doi:10.1371/journal.pone.0190735)
Supplement: S2 File — Contains data-files (each in csv format) for the 25 different language and writing systems used in our study. The name of each data file mentions the corresponding language and a serial number (e.g., “01_Arabic.csv”) in accordance with the order in which the data sets are described in the Description of the Corpora in the Supplementary Material. Each sequence is represented as a string of numbers, with each number representing a specific grapheme for that language and writing system. In addition there are key lists for each of the data-bases (in txt format for the 24 known language and writing systems and in pdf format for the undeciphered Indus database) which shows the grapheme corresponding to each number that represents them in the data-base sequences. The name of each key list mentions the corresponding language. (ZIP) [file pone.0190735.s010.zip › DatabaseKeyListV2/Keylist_Udeciphered_Indus.pdf]

001	002	003	004	005	006	007	012	013	014	015	016	017	018
191	763	231	85	41	3	5	3	25	5	6	40	76	5
019	020	025	026	027	028	029	031	032	033	034	035	036	037
5	1	2	1	6	5	2	190	507	445	153	27	5	2
039	041	042	044	045	046	047	048	049	050	051	055	056	057
1	1	2	1	2	2	1	18	5	1	1	56	9	1
058	059	060	061	062	063	064	065	066	067	068	069	070	071
1	1	204	84	3	13	11	15	10	6	1	1	35	2
072	073	074	075	080	081	082	083	084	085	090	091	092	093
16	1	1	1	2	3	2	1	1	1	168	18	1	4
094	095	097	098	099	100	101	102	103	104	105	106	107	110
2	20	20	5	1	131	9	1	4	18	1	1	1	5
111	112	113	114	115	116	117	118	119	121	122	123	125	126
11	1	1	3	1	1	3	3	1	1	2	1	50	2
127	128	129	130	131	132	133	134	135	136	137	138	139	140
3	2	1	13	1	4	1	1	1	6	17	1	1	103
141	142	143	144	145	146	147	150	151	152	153	154	155	156
1	72	4	5	2	2	1	6	80	1	2	38	3	103
157	158	159	160	161	165	166	167	168	169	170	171	172	173
1	34	6	3	11	28	1	19	3	1	1	26	1	1
175	176	177	178	179	180	190	191	192	193	194	195	200	201
18	193	4	2	2	1	10	2	2	3	1	2	2	5
202	203	204	205	206	207	208	209	215	216	217	219	220	221
4	1	6	1	1	1	1	1	9	4	2	4	435	6
222	223	224	225	226	227	228	229	230	231	232	233	234	235
13	3	1	1	36	1	1	2	4	82	10	182	8	231
236	240	241	242	243	244	250	251	252	253	255	256	257	258
19	331	12	5	12	4	8	1	7	1	120	1	9	1

260	261	262	263	264	265	266	267	268	269	270	271	272	275
8	1	2	6	2	1	1	5	6	13	1	1	3	1
276	277	278	279	280	281	285	286	287	290	291	292	293	294
1	2	2	4	1	1	4	3	2	2	1	2	1	2
295	296	297	298	299	300	301	302	303	304	305	306	307	308
1	2	13	7	1	2	2	3	1	2	1	1	2	3
309	310	311	315	316	317	318	319	320	321	322	323	324	325
2	3	2	10	2	9	13	3	21	16	3	7	2	1
326	327	328	329	330	335	336	337	338	340	341	342	343	344
7	1	1	1	1	39	11	14	1	1	2	1	1	1
345	346	347	348	350	351	352	353	354	360	361	362	363	365
3	6	10	1	44	2	3	9	12	11	2	5	1	10
367	368	370	371	372	373	374	375	380	381	382	383	384	385
2	98	6	8	1	2	14	1	6	3	16	1	26	1
386	387	388	389	390	391	392	393	394	399	400	401	402	403
1	2	57	1	243	8	3	1	1	1	432	7	7	1
404	405	406	407	408	409	410	411	412	413	415	416	417	418
1	112	14	130	5	4	2	5	1	49	165	46	8	1
420	421	422	423	425	426	427	428	429	430	435	436	440	441
5	4	6	3	1	7	1	1	1	1	63	18	48	3
442	443	444	445	450	451	452	453	454	455	456	457	458	460
6	3	2	1	2	1	1	1	1	37	6	1	1	57
461	462	465	466	467	468	469	470	471	472	473	474	475	479
1	1	1	1	8	7	1	3	2	4	2	1	2	1
480	481	482	483	484	490	491	492	493	494	495	500	501	502
33	1	33	3	13	2	18	2	1	2	25	19	29	4
503	504	505	510	511	512	513	515	516	517	518	519	520	521
79	10	1	11	11	1	2	4	4	2	8	14	282	6

525	526	527	528	530	531	532	533	534	535	540	541	542	543
8	11	62	1	11	5	1	2	1	1	17	1	1	4
544	545	546	550	551	552	554	555	556	560	561	562	563	564
3	10	1	111	5	10	11	25	6	2	2	1	1	6
565	570	571	572	573	575	576	577	578	579	585	586	590	591
15	1	1	3	1	72	1	1	4	2	58	2	203	1
592	593	595	596	597	599	600	601	602	603	604	605	610	611
10	1	46	1	9	1	5	1	1	7	3	15	2	5
615	616	617	620	621	622	623	625	626	627	628	630	631	632
62	1	77	1	20	3	1	4	7	1	1	37	2	26
633	634	635	636	637	638	639	640	641	642	645	646	647	678
1	5	1	27	6	1	4	1	1	1	24	1	2	1
679	680	681	682	683	684	685	686	687	688	689	690	692	693
18	2	5	1	3	1	3	1	5	2	2	109	79	2
694	697	698	699	700	702	703	704	705	706	707	708	709	710
1	9	1	1	565	10	11	5	200	86	1	1	3	2
711	712	713	714	715	716	717	718	719	720	721	725	726	727
18	2	1	1	1	2	54	3	1	2	1	1	1	2
728	729	731	732	733	734	735	736	740	741	742	743	744	745
1	1	1	2	1	1	1	4	1696	200	37	1	3	34
746	747	748	749	750	751	752	753	760	761	762	763	764	765
6	7	3	5	2	1	45	2	127	1	3	1	1	1
766	767	768	770	772	773	775	776	777	778	780	781	782	783
1	2	1	2	2	15	4	9	5	1	2	2	1	2
784	785	786	790	791	792	793	794	795	796	797	798	799	801
1	2	3	52	3	12	1	9	1	1	4	144	2	1
803	804	805	806	807	808	809	810	811	812	813	814	815	816
123	6	2	117	4	6	1	1	1	8	2	2	1	1

817	818	820	821	822	823	824	825	826	827	828	829	830	831
204	1	202	15	2	2	28	5	1	1	1	2	1	17
832	833	834	835	836	837	838	840	841	842	843	844	845	850
32	2	2	1	4	1	15	112	1	5	1	14	53	47
851	852	853	854	855	856	857	858	859	860	861	862	863	864
11	2	10	2	1	5	1	1	1	1	234	1	1	1
865	866	868	869	870	871	872	873	874	875	876	877	878	879
2	1	11	5	3	6	1	5	1	1	1	22	1	3
880	890	891	892	893	894	895	896	900	901	902	903	904	905
11	10	2	76	2	1	1	3	95	2	1	1	61	11
906	907	908	909	910	911	918	919	920	921	922	923	924	925
13	1	1	2	5		1	1	139	16	2	44	11	1
926	927	928	930	931	932	940	942	943	944	945	946	947	950
1	9	2	7	1	1	2	9	1	1	1	1	1	1
951	952	953	954	955	956	957	958						
1	1	1	1	1	1	2	1						
